# On the practical identifiability of a two-parameter model of pulmonary gas exchange

**DOI:** 10.1186/s12938-015-0077-6

**Published:** 2015-09-04

**Authors:** Axel Riedlinger, Jörn Kretschmer, Knut Möller

**Affiliations:** Institute of Technical Medicine, Furtwangen University, Jakob-Kienzle-Straße 17, 78054 Villingen-Schwenningen, Germany

**Keywords:** Physiological modeling, Gas exchange model, Parameter identification

## Abstract

**Background:**

Successful application of mechanical ventilation as a life-saving therapy implies appropriate ventilator settings. Decision making is based on clinicians’ knowledge, but can be enhanced by mathematical models that determine the individual patient state by calculating parameters that are not directly measurable. Evaluation of models may support the clinician to reach a defined treatment goal. Bedside applicability of mathematical models for decision support requires a robust identification of the model parameters with a minimum of measuring effort. The influence of appropriate data selection on the identification of a two-parameter model of pulmonary gas exchange was analyzed.

**Methods:**

The model considers a shunt as well as ventilation-perfusion-mismatch to simulate a variety of pathologic pulmonary gas exchange states, i.e. different severities of pulmonary impairment. Synthetic patient data were generated by model simulation. To incorporate more realistic effects of measurement errors, the simulated data were corrupted with additive noise. In addition, real patient data retrieved from a patient data management system were used retrospectively to confirm the obtained findings. The model was identified to a wide range of different *FiO*_2_ settings. Just one single measurement was used for parameter identification. Subsequently prediction performance was obtained by comparing the identified model predicted oxygen level in arterial blood either to exact data taken from simulations or patients measurements.

**Results:**

Structural identifiability of the model using one single measurement for the identification process could be demonstrated. Minimum prediction error of blood oxygenation depends on blood gas level at the time of system identification i.e. the measurement situation. For severe pulmonary impairment, higher *FiO*_2_ settings were required to achieve a better prediction capability compared to less impaired pulmonary states. Plausibility analysis with real patient data could confirm this finding.

**Discussion and conclusions:**

Dependent on patients’ pulmonary state, the influence of ventilator settings (here *FiO*_2_) on model identification of the gas exchange model could be demonstrated. To maximize prediction accuracy i.e. to find the best individualized model with as few data as possible, best ranges of *FiO*_2_-settings for parameter identification were obtained. A less effort identification process, which depends on the pulmonary state, can be deduced from the results of this identifiability analysis.

## Background

Mechanical ventilation is a life-saving intervention in intensive care, maintaining pulmonary function in critically ill patients. Appropriate ventilator settings need to be found by the clinician to ensure both sufficient oxygenation and carbon dioxide removal. Target values for arterial partial pressures of oxygen (*PaO*_2_) and carbon dioxide (*PaCO*_2_) can be reached by changing inspired oxygen fraction (*FiO*_2_) and minute ventilation (*MV*). Removal of CO_2_ and therefore *PaCO*_2_ in the patient is mainly regulated by adjusting *MV*. In critically ill patients, e.g. patients suffering from acute respiratory distress syndrome (ARDS), high levels of *FiO*_2_ and appropriate PEEP are usually necessary to ensure sufficient oxygenation. Finding the appropriate *FiO*_2_ setting follows a trial-and-error approach that may not only be tedious but also exposes the patient to the potential risk of hypoxia and hyperoxia [1–4]. Pulse oximetry allows a continuous measurement of peripheral oxygen saturation (*SpO*_2_), however this method has limitations in sensitivity and accuracy due to calibration assumptions, optical interference, and signal artifact [5]. Therefore, an invasive blood gas analysis is required at the end of each trial to evaluate the individual effect of a change in *FiO*_2_ accurately.

In mechanical ventilation therapy, both the risk of ventilator induced lung injury (VILI) and the effort to find adequate settings may be reduced if medical decision support would provide recommendations on how to adjust a patient’s settings to reach a prescribed treatment goal. In general, decision support can be divided into knowledge based (KDSS) and model based systems (MDSS). KDSS builds on rules of typical i.e. average patient behavior to represent reactions to changes of ventilator settings. In contrast, MDSS that are adapted to patient specific physiologic properties can simulate the individual reaction to changes in therapy settings. Using the inverse model, MDSS therefore suggests individualized ventilator settings by evaluating the approximated physiology of the patient.

Parameters of a model contain compact information about the individual patient state and dynamics once they are quantified in a parameter identification process (PIP). Parameter identification requires information from patient measurements often obtained during certain clinical maneuvers. Success and robustness of the PIP strongly depends on the properties of the model to reflect the required dynamics of the patient, the signal quality and amount of data available at the bedside. As the identified parameters are used for forward calculation of the model equations, they directly influence prediction performance of the model. While using multiple and even redundant measurements helps compensating noise induced errors, measurement efforts and applying the necessary maneuvers should not interfere with clinical processes. Thus, those measurements should be kept to the minimum necessary to ensure a robust PIP.

Models of pulmonary gas exchange are able to predict the effect of *FiO*_2_ and *MV* on *PaO*_2_ and *PaCO*_2_ in the patient. One-parameter models [6, 7] usually only consider shunt, i.e. the amount of venous blood that is mixed with the oxygenated blood, to describe a patient’s oxygenation status and to predict the effect of an increase of *FiO*_2_ on *PaO*_2_. However, using only one parameter to describe gas exchange impairments fails at low *FiO*_2_ when mismatches between alveolar ventilation (*V̇*) and perfusion (*Q*) occur. Several studies [8, 9] have come to the conclusion that *PaO*_2_*/FiO*_2_ ratio, usually used to categorize lung impairment, changes with *FiO*_2_. Thus, besides shunt, mathematical models of gas exchange should either include a parameter to describe oxygen diffusion limitation [10, 11] or a parameter to characterize $\dot{V}/Q$ mismatch [9, 12–14]. Latter have shown to reproduce measurements at different oxygenation levels with sufficient accuracy compared to more complex models or MIGET measurements [15]. A two-parameter model of pulmonary gas exchange including shunt and $\dot{V}/Q$ mismatch has previously been published by Kjaergaard et al. [12]. Karbing et al. [16] evaluated this model with data of severely ill intensive care patients. The model has been found to be identifiable with four pulse oxymetric (*SpO*_2_) measurements at different levels of *FiO*_2_ and one blood gas analysis (BGA) providing *PaO*_2_ and *PaCO*_2_ together with the acid–base parameters *pH*, base excess (*BE*) and the hemoglobin concentration (*cHb*) as well as the end-tidal gas fractions of oxygen (*FetO*_2_) and carbon dioxide (*FetCO*_2_). Although systems have been built to perform the necessary measurements in 10–15 min [17], lowering the number of measurements required for identification and therefore minimizing the required time and effort is highly relevant. Therefore we investigated if the number of measurements that are necessary to identify the model can be reduced to one *FiO*_2_-setting. Additionally, we evaluated the influence of the chosen level of *FiO*_2_ during model identification.

## Methods

### Gas exchange model with $\dot{V}/Q$-mismatch and shunt

**Fig. 1:**
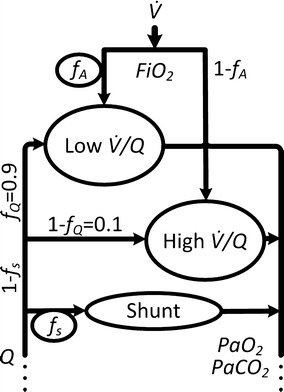
Schematic representation of the gas exchange model. The model consists of two alveolar compartments, one with low and one with high ratio between ventilation $\dot{V}$ and perfusion *Q* respectively. Blood flow is distributed among the shunt *f*_*s*_ and the two alveolar compartments. The low $\dot{V}/Q$-compartment is perfused with a fixed fraction *f*_*Q*_ of 90 % of the non-shunted blood and ventilated with fraction *f*_*A*_. *FiO*_2_ describes the fraction of oxygen in inspired air. *PaO*_2_ and *PaCO*_2_ are arterial partial pressures of oxygen and carbon dioxide respectively

The mathematical model of human pulmonary gas exchange consists of two alveolar compartments that are perfused and ventilated and one shunt compartment that is perfused but not ventilated. The alveolar compartments are separated into a compartment with high $\dot{V}/Q$-ratio and a compartment with low $\dot{V}/Q$-ratio. This allows the consideration and simulation of limitations in gas exchange for both oxygen (O_2_) and carbon dioxide (CO_2_) concentrations in blood. Shunt, i.e. the fraction of venous blood not participating in gas exchange, is quantified by model parameter *f*_*s*_ multiplied with blood flow *Q*. 90 % of the non-shunted blood ((1 − *f*_*s*_)*Q) is distributed to the low $\dot{V}/Q$ compartment, 10 % of the non-shunted blood is delivered to the high $\dot{V}/Q$ compartment. Model parameter *f*_*A*_ represents the fraction of alveolar ventilation *V̇*_*A*_ that reaches the low $\dot{V}/Q$ compartment. Figure 1 shows the model structure of the pulmonary gas exchange model.

The model assumes equilibrium in blood gas concentrations as well as constant alveolar ventilation and perfusion without separating ventilation into phases of inspiration and expiration. Model inputs are *FiO*_2_ as well as end-tidal blood gas fractions of oxygen (*FetO*_2_) and carbon dioxide (*FetCO*_2_), respectively. Inspired carbon dioxide is set to 0. Tidal volume *V*_*tid*_ and respiratory frequency *f*_*R*_ are assumed to be constant during simulation and are provided as additional model inputs. Model outputs are the resulting arterial blood gas parameters *PaO*_2_ and *PaCO*_2_.

Alveolar ventilation *V̇*_*A*_ is calculated from *f*_*R*_ and the difference between tidal volume *V*_*tid*_ and the anatomic dead space volume *V*_*ds*_:

1 $$\dot{V}_{\text{A}} = f_{\text{R}} * \left( {V_{\text{tid}} - V_{\text{ds}} } \right)$$

*FetO*_2_ and *FetCO*_2_ are composed of alveolar gas fractions *FAO*_2_ and *FACO*_2_ in both compartments, such that

2 $${Fet}_{x} = (1 - f_{A} )*{FA}_{{{x},1}} + f_{A} *{FA}_{{{x},2}}$$

Index *x* represents O_2_ and CO_2_ in Eq. 2 and in all following equations. Index 1 refers to the alveolar compartment with high $\dot{V}/Q$, while index 2 denotes the low $\dot{V}/Q$ compartment. Oxygen consumption *V̇*_*O*2_ and carbon dioxide production *V̇*_*CO*2_ are derived from alveolar air flow to each of the compartments and the difference between inspired and alveolar gas fractions as described in Eqs. (3) and (4):

3 $$\dot{V}_{x,1} = \left( {1 - f_{A} } \right) *\dot{V}_{A} * (Fi_{x} - FA_{x,1} )$$

4 $$\dot{V}_{{{x},2}} = f_{A} *\dot{V}_{A} *({Fi}_{x} - {FA}_{{{x},2}} )$$

Capillary blood gas concentrations *Cc*_*x*_ are derived from alveolar gas fractions using O_2_ and CO_2_ dissociation curves [18, 19] (T—temperature):

5 $$Cc_{x} = blood(FA_{x} ,pH,T,cHb)$$

Venous blood gas concentrations are then calculated as described in Eqs. (6) and (7).

6 $${Cv}_{{{x},1}} = {Cc}_{{{x},1 }} - \dot{V}_{{{x},1 }} / ({Q} * \left( {1 - {f}_{s} } \right) * 0.1)$$

7 $${Cv}_{{{x},2}} = {Cc}_{{{x},2}} - \dot{V}_{{{x},2}} / ({Q} * \left( {1 - {f}_{s} } \right) * 0.9)$$

Cardiac output *Q* is measured at the bedside or estimated from the patient’s body surface area.

*FAO*_2_ and *FACO*_2_ are solved numerically for a given measured *FetO*_2_ and *FetCO*_2_ with the condition that venous concentration in both compartments is equal. Finally, arterial blood gas concentrations *CaO*_2_ and *CaCO*_2_ are calculated as:

8 $${Ca}_{x} = {Cc}_{{{x},1}} *\left( {1 - {f}_{s} } \right)* 0.1 + {Cc}_{{{x},2}} *\left( {1 - {f}_{s} } \right)*0.9 + {Cv}_{x} *{f}_{s}$$

Arterial partial pressures of oxygen and carbon dioxide are then calculated from the reversed dissociation curves:

9 $$Pa_{x} = blood(Ca_{c} ,pH,T,cHb)$$

### Model simulation

**Fig. 2:**
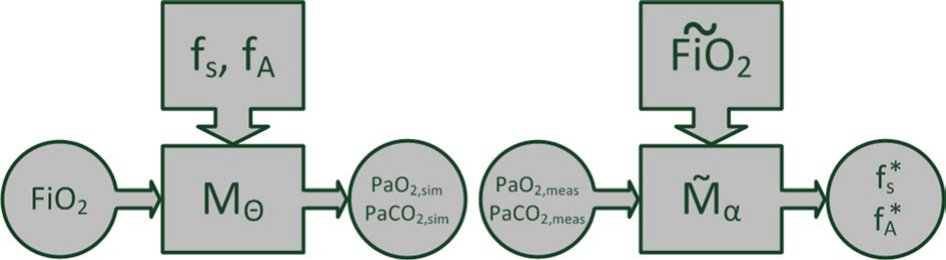
Flowcharts of model simulation (*left*) and model identification (*right*). In model simulation, blood gas levels are calculated with respect to *FiO*_2_. Model parameters *f*_*s*_ and *f*_*A*_ are known. Vector Θ summarizes parameters as well as other physical constants necessary for model calculation. Model identification process $\tilde{M}_{\alpha }$ minimizes an objective function for measured blood gas levels at a specific $\widetilde{FiO}_{2}$. Identified parameters are *f*_s_^*^ and *f*_A_^*^

Forward calculation of the model equations is termed as model simulation. The flowchart of the model simulation process *M*_*Θ*_ is depicted in Fig. 2 on the left. Vector *Ψ* summarizes physical constants for measurements needed for model simulation:

$$\Psi = \left( {{MV},{V}_{tid} ,{V}_{ds} ,{Q},{cHb},{pH},{T},{FetO}_{ 2} ,{FetCO}_{ 2} } \right).$$

Here, minute ventilation MV is calculated as:

10 $$MV = V_{tid} *f_{R}$$

Vector *Θ* includes model parameters *f*_*s*_ and *f*_*A*_ as well as *Ψ*:

$$\varTheta = (f_{s} , f_{A} , \varPsi ).$$

Output values *PaO*_2*,sim*_ and *PaCO*_2*,sim*_ are calculated depending on *Θ* and *FiO*_2_:

$$\left( {PaO_{2,sim} , PaCO_{2,sim} } \right) = M_{\varTheta } (FiO_{2} ).$$

### Model identification

Parameters that need to be identified in the presented model are shunt fraction (*f*_*s*_) and the fraction of alveolar ventilation that is distributed to the alveolar compartment with low $\dot{V}/Q$-ratio (*f*_*A*_). The process of model identification $(\tilde{M}_{a})$ is shown on the right of Fig. 2. $(\tilde{M}_{a})$ is a minimization process of an objective function. $\widetilde{FiO}_{2}$, i.e. the level of inspired oxygen at the time of the measurement, as well as the other constant physiological values required during identification, are represented in vector *α*:

$$\alpha = (\widetilde{FiO}_{2} , \varPsi ).$$

$PaO_{2,meas}$ and $PaCO_{2,meas}$ are the measured blood gases obtained at a specific condition described by *α*. They are used to determine *f*_s_^*^ and *f*_A_^*^ that best reproduce the measurements in the forward model:

$$\varTheta^{*} = \left( {f_{s}^{*} , f_{A}^{*} , \varPsi } \right) = \tilde{M}_{\alpha } (PaO_{2,meas} , PaCO_{2,meas} ).$$

Parameter identification was performed by minimizing the sum of the squared error (*SSE*) between measured (*meas*) and predicted (*pred*) partial blood gas pressures in arterial blood:

$$\left( {PaO_{2,pred} ,PaCO_{2,pred} } \right) = M_{{\varTheta^{*} }} (\widetilde{FiO}_{2} )$$

11 $$SSE = (PaO_{2,meas} - PaO_{2,pred} )^{2} + 3 * (PaCO_{2,meas} - PaCO_{2,pred} )^{2}$$

The weighting factor of 3 for *PaCO*_2_ was chosen to avoid imbalanced influence of *PaO*_2_ data on the identification process, as dimension of *PaCO*_2_ is approximately three times smaller than *PaO*_2_. Minimization of the above described objective function was carried out using *fminsearchbnd* in MATLAB (R2012a, The Mathworks, Natick, MA, USA). *fminsearchbnd* is distributed under the BSD license and is based on *fminsearch*, the MATLAB function that employs the Nelder-Mead simplex search method [20]. According to [21] a shunt of 50 % and above leads to increases in *FiO*_2_ having no effect on *PaO*_2_. Additionally, *f*_*A*_ values above 0.9 lead to a swap of the high $\dot{V}/Q$-compartment with the low $\dot{V}/Q$-compartment, essentially mirroring $\dot{V}/Q$-values of *f*_*A*_ below 0.9. Thus parameter constraints for {*f*_*s*_, *f*_*A*_} were set as nonnegative lower boundaries LB = {0, 0} and as upper boundaries UB = {0.5, 0.9}.

Structural identifiability of the model using multiple measuring points was shown in a previous report [22]. Initial *fs* was set to 0.2, which showed to lead to the global minimum of the objective function in all test cases. Initial *f*_*A*_ was set arbitrarily to 0.5 to start the minimization process at a certain initial mismatch between ventilation and perfusion. Constant patient state for the time of model prediction is assumed.

### Model prediction

Prediction of blood gas levels depending on *FiO*_2_ is done using forward calculation of the model M_Θ*_ with Θ^*^ = (*f*_s_^*^, *f*_A_^*^, *Ψ*):

$$\left( {PaO_{2,pred} , PaCO_{2,pred} } \right) = M_{{\varTheta^{*} }} (FiO_{2} ).$$

### Data

We have employed both simulated and recorded real patient data to evaluate how well the described model is identifiable with data obtained at one single *FiO*_2_ level.

*Simulated data* The same two-parameter model of pulmonary gas exchange was used to create experimental data. It allows calculating the impact of noise in the data because the correct results for parameter identification are known a priori. Twelve classes of patient data sets have been generated, that differ in the parameter combinations of *f*_*s*_ and *f*_*A*_. Those have been chosen to represent different stages of pulmonary disease. Model parameters used for data generation, resulting *FiO*_2_*/PaO*_2_-ratios and the classifications of pulmonary impairment [24] are listed in Table 1. BGA and physiological standard values of an adult man were used for data generation. These physical constants are listed and explained in Table 2.

**Table 1:** Parameters used for simulation of patient data

Patient no. (j)	*f*_*S*_	*f*_*A*_	*High* $\dot{V}/Q$	*Low* $\dot{V}/Q$	*PaO*_2_ */FiO*_2_-ratio	Classification of impairment
1	0.05	0.90	1.15	1.15	412	Healthy
2	0.10	0.70	3.64	0.94	303	Mild
3	0.15	0.70	3.85	1.00	227	Mild
4	0.20	0.70	4.09	1.06	169	Moderate
5	0.20	0.50	6.82	0.76	162	Moderate
6	0.25	0.50	7.27	0.81	123	Moderate
7	0.25	0.30	10.18	0.48	114	Moderate
8	0.30	0.50	7.79	0.87	95	Severe
9	0.30	0.30	10.91	0.52	89	Severe
10	0.35	0.50	8.39	0.93	74	Severe
11	0.35	0.30	11.75	0.56	72	Severe
12	0.35	0.15	14.27	0.28	70	Severe

Parameter values for shunt fraction *f*_*s*_ and fraction of ventilation distribution *f*_*A*_ as well as the resulting $\dot{V}/Q$-ratios are shown. Classification of pulmonary state is based upon $\dot{V}/Q$-ratio and *PaO*_2_/*FiO*_2_-ratio [24]

**Table 2:** Constants used for generation of data sets

Item	Abbreviation	Value	Unit
Minute volume	*MV*	6	l/min
Tidal volume	*Vtid*	0.5	l
Dead space volume	*Vds*	0.15	l
Cardiac output	*Q*	5.5	l/min
Hemoglobin concentration	*cHb*	140	g/l
Alveolar pH value	*pH*	7.4	–
Base excess	*BE*	0	mmol/l
Temperature	*T*	37	°C
Respiratory quotient	*RQ*	0.8	–

Physiological constants of an adult man were used for generation of patient data sets

More formally, we define twelve patient classes by the model parameters

$$\varTheta_{j} = \left( {f_{{s_{j} }} , f_{{A_{j} }} , \varPsi } \right)\quad {\text{with}}\,\,1 \le j \le 8.$$

For each of the twelve patient classes, 1000 simulated measurements equidistant between *FiO*_2_ of 21 % and 100 % were determined. Depending on *FiO*_2_ settings, model simulation led to

$$\left( {PaO_{2,sim}^{j} , PaO_{2,sim}^{j} } \right) = M_{{\varTheta_{j} }} (FiO_{2} ).$$

Measuring *PaO*_2_ and *PaCO*_2_ via blood gas samples drawn from the arterial line is the current gold standard in clinical practice [25, 26], while measuring arterial oxygen saturation via pulse oximetry is accurate within ±2 % of the true value [27]. Thus, to account for measurement noise that would be present in a real setting, both *PaO*_2*,sim*_ and *PaCO*_2*,sim*_ data were superimposed with uniformly distributed noise in a range of ±5 %:

$$\left( {PaO_{2,meas}^{j} , PaO_{2,meas}^{j} } \right) = noise\left( {PaO_{2,sim}^{j} , PaO_{2,sim}^{j} } \right).$$

The quality of system identification was assessed with a test set (ts) of 17 distinct $FiO_{2}^{ts}$ values ranging from 21 to 100 % in steps of 5 %:

$$\left( {PaO_{{2,sim_{i} }}^{ts} , PaCO_{{2,sim_{i} }}^{ts} } \right) = M_{\varTheta } (FiO_{2i}^{ts} )\quad {\text{for}}\,\,1 \le i \le 17.$$

Real patient data: Two real patient data sets were used for the plausibility check of the results obtained from the theoretical analysis. Real patient data including at least four blood gas measurements at different *FiO*_2_ settings in mandatory ventilation mode were retrieved from a patient data management system of the university medical centre in Kiel [7]. Two data sets, a mild (Pat R1) and a critically ill patient (Pat R2), met those demands. The recorded levels of *FiO*_2_ were applied on a therapeutical basis, thus not systematically in the context of a clinical trial. The data sets included measurements of *PaO*_2_, *PaCO*_2_, *f*_*R*_, *Q*, *V*_*tid*_, *V*_*ds*_, *cHb*, *pH*, *T* and *FetCO*_2_ at each of the applied *FiO*_2_ levels. Patient data did not include *FetO*_2_ measurements, thus *FetO*_2_ was approximated from:

12 $$FetO_{2} = FiO_{2} - \frac{{FetCO_{2} }}{RQ}$$

Here, the respiratory quotient *RQ* was assumed to be 0.8. As with the simulated data sets, initial conditions of *{fs, fA}* for parameter identification were set to {0.2, 0.5}.

### Analysis of structural identifiability

To verify structural identifiability of a model system, uniqueness of the solution of parameter identification has to be proven. The simplicity of the two-parameter model allows a numerical calculation and two-dimensional visualization of the objective function. The *SSE* is calculated and plotted for different parameter combinations to visualize the contour of the error surface. A single global minimum of the objective function indicates structural identifiability of the model.

Structural identifiability of the model using one measurement point at one $\widetilde{FiO}_{2}$ level was analyzed with the synthetic as well as the two real patient data sets. The error surfaces (*SSE*) were plotted as a function of model parameters *f*_*s*_ and *f*_*A*_ with a resolution of 90 × 90.

### Evaluation of quality of fit

Besides quantity, quality of measurements used for model identification is essential for the accuracy of parameter identification. To verify practical identifiability, the influence of measuring errors on identification behavior of the model system was evaluated using the 1000 virtual measurements ($PaO_{2,meas}^{j} , PaCO_{2,meas}^{j}$) in each of the eight classes of virtual patients.

For every patient class the gas exchange model was identified with only one single of the 1000 noisy measurements. Please note that each of those measurements belongs to one *FiO*_2_ setting:

$$\varTheta_{j}^{*} = \left( {f_{{s_{j} }}^{*} , f_{{A_{j} }}^{*} , \varPsi } \right) = \tilde{M}_{\alpha } (PaO_{2,meas}^{j} , PaCO_{2,meas}^{j} )\quad {\text{for}}\,\,1 \le j \le 8.$$

With the identified parameters *f*_s_^*^ and *f*_A_^*^, the evaluations $PaO_{{2,pred_{i} }}^{ts}$ as well as $PaCO_{{2,pred_{i} }}^{ts}$ were calculated for all 1 ≤ i ≤ 17 *FiO*_2_-values in the test set:

$$\left( {PaO_{{2,pred_{i} }}^{ts} , PaCO_{{2,pred_{i} }}^{ts} } \right) = M_{{\varTheta_{j}^{*} }} (FiO_{2i}^{ts} )\quad {\text{for}}\,\,1 \le i \le 17.$$

Predictive performance for blood oxygenation as well as partial pressure of carbon dioxide was evaluated by comparing $PaO_{2,sim}^{ts}$ and $PaCO_{2,sim}^{ts}$, i.e. the values of the original simulation set, with $PaO_{2,pred}^{ts}$ and $PaCO_{2,pred}^{ts}$ respectively. Mean deviations were calculated with

$$\overline{\Delta PaO}_{2} = \frac{1}{17} \mathop \sum \limits_{i = 1}^{17} \Delta PaO_{{2_{i} }} = \frac{1}{17} \mathop \sum \limits_{i = 1}^{17} \left| {PaO_{{2,pred_{i} }}^{ts} - PaO_{{2,sim_{i} }}^{ts} } \right|$$

and

$$\overline{\Delta PaCO}_{2} = \frac{1}{17} \mathop \sum \limits_{i = 1}^{17} \Delta PaCO_{{2_{i} }} = \frac{1}{17} \mathop \sum \limits_{i = 1}^{17} \left| {PaCO_{{2,pred_{i} }}^{ts} - PaCO_{{2,sim_{i} }}^{ts} } \right|.$$

For statistical evaluation, the 1000 $\widetilde{FiO}_{2}$ levels, ranging from 21 % to 100 %, were divided into eight clusters (21–30, 30–40,…, 90–100 %), each cluster containing 125 values. Mean and standard deviations of $\overline{\Delta PaO}_{2}$ for each cluster were calculated.

### Verification of results with patient data

Two real patient data sets were used to confirm the findings obtained with simulated data. Identification was conducted at each *FiO*_2_ value that was recorded in the particular patient. Predictive performance was evaluated by comparing measured and predicted *PaO*_2_ and *PaCO*_2_ at all four recorded *FiO*_2_ levels:

$$\Delta PaO_{{2_{n} }} = \left| {PaO_{{2,pred_{n} }}^{ts} - PaO_{{2,meas_{n} }}^{real} } \right|$$

$$\Delta PaCO_{{2_{n} }} = \left| {PaCO_{{2,pred_{n} }}^{ts} - PaCO_{{2,meas_{n} }}^{real} } \right|\quad {\text{with}}\,\,n = \, 6\,\,{\text{for}}\,\,{\text{Pat}}\,\,{\text{R}}1\,\,{\text{and}}\,\,n = 4\,\,{\text{for}}\,\,{\text{Pat}}\,\,{\text{R}}2.$$

## Results

### Visualizing the objective function

**Fig. 3:**
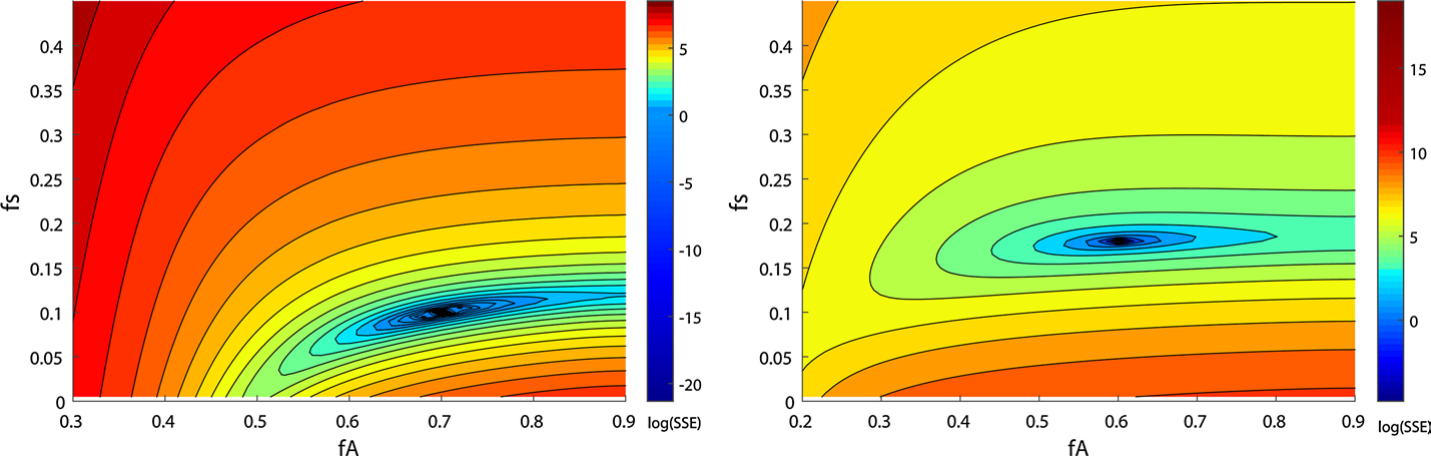
Contour plot of objective functions (*SSE*) with logarithmic scale. log(SSE) was plotted with respect to parameters f_s_ and f_A_. *Left* SSE for simulated data set no. 5. The global minimum was located at {*f*_*s*_, *f*_*A*_} = {0.1, 0.7}, the parameter values set for data generation. *Right* *SSE* for real patient data Pat R1. Global minimum was located at {*f*_*s*_, *f*_*A*_} = {0.17, 0.6}

Figure 3 visualizes the contour of the objective function evaluated at one single measuring point. Figure 3a shows synthetic patient data, while Fig. 3b is devoted to real patient data. The contour lines (SSE) are scaled logarithmically to improve visibility of the minimum. For all analyzed data sets, a single global minimum in the error surface as a function of the model parameters *f*_*s*_ and *f*_*A*_ could be detected. The parameter combination leading to the global minimum was in agreement with the parameters used for data generation. Within accuracy of numerical representation (double), the *SSE* value was zero at the minimum. The global minimum is inside a narrow and flat valley, parallel to the axis of parameter *f*_*A*_. A similar type of shape of the objective function could be shown for the analysis of both simulated and real data.

### Prediction of *PaCO*_2_ and *PaO*_2_ depending on *FiO*_2_

**Fig. 4:**
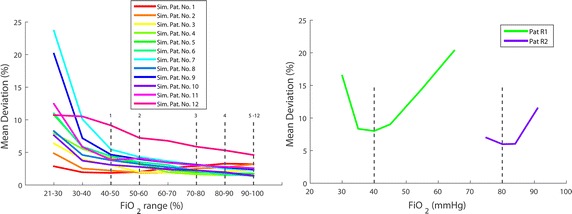
*Left* clustered mean deviation of $\overline{\Delta PaO}_{2}$ over *FiO*_2_ for simulated data. Deviation of prediction of *PaO*_2_ depends on *FiO*_2_ range used for model identification. *Broken lines* show respective minima for the different data sets (*numbered*). Minimum is located at a higher *FiO*_2_ range for data representing a higher pulmonary distress. *Right* mean of *ΔPaO*_2_ over *FiO*_2_ for real patient data. Deviation of prediction of *PaO*_2_ varies with the *FiO*_2_ level used for model identification. The location of the minimum depends on patients’ pulmonary state. Mean deviation of *ΔPaO*_2_ was less than 10 % at the minimum for both data sets

Over all tested data sets, mean $\overline{\Delta PaCO}_{2}$ was 2.4 % (1.3 mmHg) of the true value with a standard deviation of ±1.6 % (±0.8 mmHg). Figure 4a shows mean deviation of $\overline{\Delta PaO}_{2}$ with respect to *α*. *PaO*_2_ prediction was less accurate when identification data were recorded at low *FiO*_2_ levels, especially for data sets representing pulmonary impairment. For data set 1 (healthy lung) minimal $\overline{\Delta PaO}_{2}$ was achieved for 40 % < *FiO*_2_ < 50 % whereas model identification with data set 12 (severely impaired lung) shows the smallest $\overline{\Delta PaO}_{2}$ for α = (100 %, Ψ). In the minima, the model was able to reproduce *PaO*_2_ of all of the simulated patient data sets with a mean deviation of less than 2.5 % (<2.5 mmHg) of the true value with a standard deviation of less than 3 % (<2 mmHg). In Fig. 4a, the minima are marked with vertical lines and the respective patient numbers.

Mean deviation of *ΔPaO*_2_ for real patient data sets is shown in Fig. 4b. Predictions with model parameters being identified at low and high *FiO*_2_ show higher deviations from measured values than for identification at medium *FiO*_2_ levels. Mean deviation of *ΔPaO*_2_ was less than 10 % (8 % or 5.8 mmHg for Patient R1 and 6 % or 4.6 mmHg for Patient R2) at the minimum. The best performance of *PaO*_2_ prediction was found for α = (40 %, Ψ) (Pat R1) and α = (80 %, Ψ) (Pat R2) respectively.

**Fig. 5:**
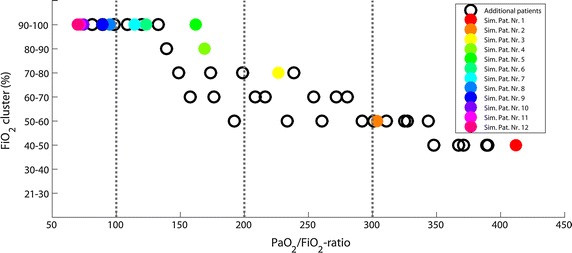
*FiO*_2_ cluster of minima of $\overline{\Delta PaO}_{2}$ mean deviation with respect to *PaO*_2_ */FiO*_2_-ratio for simulated data. *Colored markers* show the minima of the eight simulated data sets of Table 1 (numbered). Additional data were generated to visualize the curve progression and to confirm the findings (*black markers*). Minimum of $\overline{\Delta PaO}_{2}$ mean deviation is shifted to a higher *FiO*_2_ cluster for increasing severity of pulmonary impairment (lower *PaO*_2_ */FiO*_2_-ratio)

Figure 5 summarizes the *FiO*_2_ levels leading to a minimum of mean deviation of $\overline{\Delta PaO}_{2}$ for simulated data sets (colored markers). 32 additional simulated data sets (black markers) were generated to illustrate the relation between optimal *FiO*_2_ and pulmonary impairment more precisely. Location of the minimum was shifted to a higher *FiO*_2_ cluster with increasing pulmonary impairment, i.e. decreasing *PaO*_2_/*FiO*_2_-ratio.

## Discussion

Using mathematical models for decision support in clinical practice requires high level of safety and accuracy of the model predictions. Furthermore, measuring effort for model identification, i.e. identification of patient specific parameters, should be kept to a minimum.

Identification of the two-parameter model of human pulmonary gas exchange requires data from arterial blood gas analysis and photoplethysmographic saturation measurement. The model has previously been applied with four measurements at different levels of *FiO*_2_. To reduce the effort required in clinical practice to identify gas exchange models it was investigated if a reduced number of *FiO*_2_ levels for data acquisition is sufficient.

Structural identifiability of the model applying a single identification data point was examined in this study with both simulated and real patient data sets. One single global minimum of the objective function is an indication for structural identifiability of the model. Without noise in the data, one single blood gas measurement is sufficient for robust model identification.

The effect of a change in *FiO*_2_ on the concentration of carbon dioxide in arterial blood is negligible. *PaCO*_2_ data could be reproduced by the model with high accuracy. Prediction error of *CO*_2_ data was below the noise level of ±5 % for all data sets. However, measuring errors may decrease the accuracy of parameter identification and therefore model prediction of *PaO*_2_, especially if identification is based on only one measurement. Results show that the gas exchange model with shunt and $\dot{V}/Q$-mismatch is able to fit both the synthetic and the real patient data with good accuracy, as already presented in former work [16]. Oxygenation data of all data sets could be reproduced by the model with a mean deviation below 10 % in spite of measuring errors in the identification data. However, when identifying the model with noisy data, the *FiO*_2_ setting influences the prediction accuracy of *PaO*_2_. This influence was therefore examined to find a guideline how to choose an appropriate *FiO*_2_ level for data acquisition in the identification process.

The identification processes of both simulated and real patient data sets representing a variety of different disease states showed surprisingly similar results. It could be pointed out that accuracy of model prediction of blood gas concentration is related to the *FiO*_2_ setting when recording identification data. The optimum *FiO*_2_ level depends on the level of pulmonary impairment whereupon *FiO*_2_ should be increased with increasing severity of pulmonary impairment. In severely ill patients, oxygenation of arterial blood is inhibited, thus higher *FiO*_2_ levels are required in order to achieve adequate *PaO*_2_ levels.

**Fig. 6:**
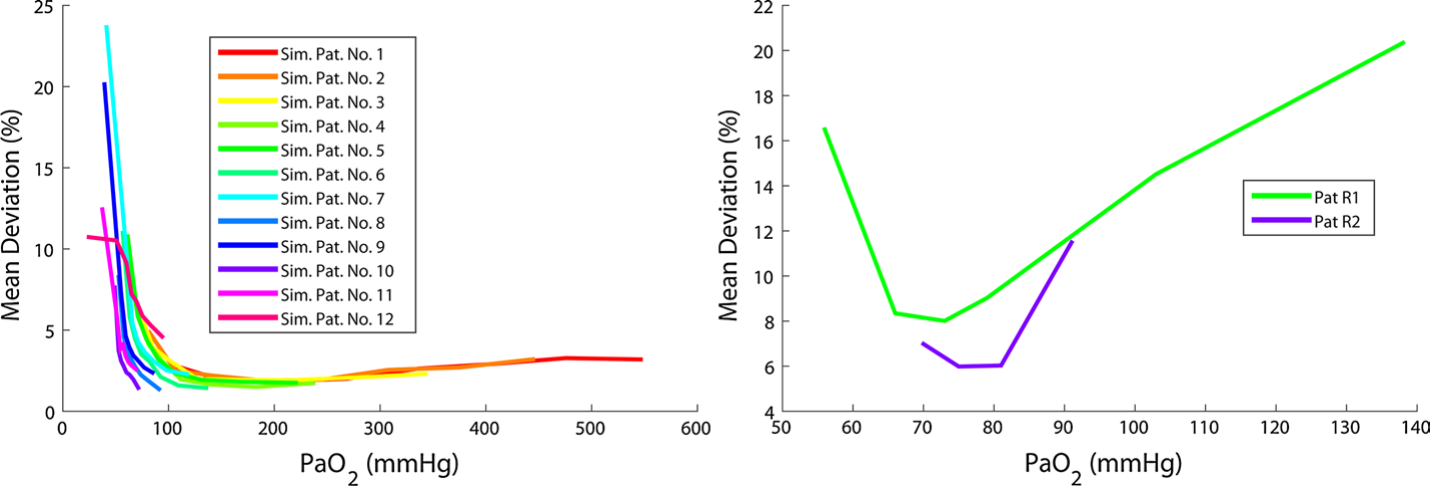
*Left* clustered mean deviation of $\overline{\Delta PaO}_{2}$ over $PaO_{2,meas}^{j}$ for simulated data. Minimum in prediction error of *PaO*_2_ data is in the range of 150–200 mmHg for data sets 1–7. Because of the high severity of pulmonary impairment, data sets 8–12 do not achieve this oxygenation range, even when a *FiO*_2_ of 100 % is applied. *Right* mean of *ΔPaO*_2_ over $PaO_{2,meas}^{real}$. Real patient data tested in our study confirm the curve progression of the study with the simulated data. The best prediction performance is shown for identification in the *PaO*_2_ range of 70–80 mmHg

Figure 6a shows the mean deviation of $\overline{\Delta PaO}_{2}$ with respect to $PaO_{2,meas}^{j}$. It could be observed that a minimal prediction error is achieved for $PaO_{2,meas}^{j}$ in the range of 150–200 mmHg for the entire simulated data sets. Identification at higher levels leads to a small increase of mean deviation. However, mean deviation of $\overline{\Delta PaO}_{2}$ was found to be still below 5 % for all tested patient cases. Identifying the model with *PaO*_2_ levels of less than 100 mmHg is potentially leading to an increase in both mean and standard deviation of model prediction. Because of the high severity of pulmonary impairment, data sets 7 and 8 do not achieve a *PaO*_2_ of 100 mmHg, even when a *FiO*_2_ of 100 % is applied. Patients with such high pulmonary impairment have to be ventilated using the highest *FiO*_2_ possible to achieve a sufficient oxygenation.

Mean deviation of *ΔPaO*_2_ over $PaO_{2,meas}^{real}$ is shown in Fig. 6b. Both curves representing real data confirm the results of the analysis with simulated data, but minimum mean deviations were found for a *PaO*_2*,meas*_ of 73 mmHg and 81 mmHg respectively.

When only one measuring point is used for model identification, success of parameter identification obviously depends on the level of *PaO*_2_ at the particular time of the measurement. Low *PaO*_2_ levels bear the risk of large influence of measuring errors in identification data. In low *PaO*_2_ levels, even small changes in *PaO*_2_ used for identification may lead to an overestimation of shunt fraction *f*_*s*_ and therefore to a smaller increase of *PaO*_2_ for higher *FiO*_2_ levels compared to correct data. In severely ill patients, this effect is more prominent than in patients with less pulmonary distress.

*PaO*_2_ levels suggested to be optimal for identification of the model may be not achievable in patients with severe pulmonary impairment. Here, identification at an *FiO*_2_ of 100 % was shown to achieve predictions with highest accuracy.

In the presented work, we have investigated a *FiO*_2_ range 21–100 %. To separate effects of shunt from those caused by low $\dot{V}/Q$-ratio, subatmospheric oxygen levels should be considered as well. However, the intended use of the applied model and the presented routine of identifying the model parameters with only one blood gas measurement are in a critical care environment where such oxygen levels are not applied.

Karbing et al. [16] have previously presented a three-parameter extension of the model used in this work which uses an adjustable distribution of non-shunted blood among the alveolar compartments. This model shows to be superior in terms of reproducing *PaCO*_2_ especially in patients with $\dot{V}/Q$-ratios below 0.27. The presented work however focuses mainly on finding the optimal calibration point of *FiO*_2_, which has only a minor effect on *PaCO*_2_. Nevertheless, investigating the structural identifiability and the possibility of using only one measurement set to also identify the three-parameter model should be considered in future work.

The model of gas exchange applied in the presented study includes assumptions such as steady state conditions of blood gases and constant alveolar ventilation and perfusion. Thus, tidal breathing as is the reality in humans is not considered. Those assumptions present shortcomings compared to the reality those models try to reproduce [28]. Several models including tidal breathing have been presented in the past [28–30] however continuous measurement of blood gases in combination with continuous measurements of gases in inspired and expired air is not routinely available at the bedside at this moment. Thus clinical applications are currently limited to models assuming continuous ventilation and perfusion as well as equilibrated blood gases. Still, Karbing et al. [16] have shown previously that the applied model is well capable of reproducing patient data for a wide range of lung impairments and that the model can thus be used in a clinical environment as a prediction tool.

Model based decision support in clinical practice implies that the mathematical model is identifiable with a minimum of measuring effort. We could show that the two-parameter gas exchange model with shunt and $\dot{V}/Q$-mismatch is structural identifiable with only one single blood gas measurement. Using only one single measurement, possible measuring errors are not averaged. However, simulation results show that model based prediction of blood gases for different *FiO*_2_ is possible with a mean prediction error below 10 % for a maximum measuring error of 5 %. Simultaneously, we could determine the range of *PaO*_2_ level where prediction error is minimized for data representing a wide range of different pulmonary states. Our work provides scientific findings in developing a robust parameter identification process for the gas exchange model with low measuring effort. For a given accuracy of the blood gas measurements used for identification it will be possible to estimate the accuracy of the model prediction of blood gases.

This study is faced with the limitation that only two real patient data sets were available to confirm the findings from the study with simulated data. Furthermore, the real data were not from a systematic patient study, but retrieved from a patient data management system, giving no information about the interventions of the clinicians between the measurements. Therefore, a change in the patients’ pulmonary state cannot be excluded. A systematic study with a higher number of mechanically ventilated patients is necessary to consolidate our findings.

## Conclusions

The study showed that the identification point has a significant impact on the predictive performance of the presented gas exchange model. Measuring errors, i.e. noise in identification data, could lead to prediction errors when only one measurement is applied. A combination of simulated and real patient data provides a valuable tool in determining the optimal identification point where influence of measurement errors is minimal.

## List of abbreviations

ARDS: acute respiratory distress syndrome
*BE*: base excess
BGA: blood gas analysis
*Cc*: capillary concentration
*cHB*: concentration of hemoglobin
*CO*_2_: carbon dioxide
*Cv*: venous concentration
*f*_*A*_: fraction of ventilation distribution
*FA*: alveolar fraction
*Fet*: end-tidal fraction
*FiO*_2_: inspired oxygen fraction
*f*_*Q*_: fraction of perfusion distribution
*f*_*R*_: respiratory frequency
*f*_*s*_: shunt fraction
KDSS: knowledge based decision support system
LB: lower boundaries
MBSS: model based decision support system
MIGET: multiple inert gas elimination technique
MV: minute ventilation
*O*_2_: oxygen
*Pa*: arterial partial pressure
PIP: parameter identification process
*Q*: cardiac output
*RQ*: respiratory quotient
*SpO*_2_: peripheral oxygen saturation
*SSE*: sum of the squared errors
*T*: patient temperature
UB: upper boundaries
*V̇*: ventilation
*V̇*_*A*_: alveolar ventilation
*V̇*_*CO*2_: carbon dioxide production
*V*_*ds*_: dead space volume
VILI: ventilator induced lung injury
*V̇*_*O*2_: oxygen consumption
*V*_*tid*_: tidal volume
